# A new model of sperm nuclear architecture following assessment of the organization of centromeres and telomeres in three-dimensions

**DOI:** 10.1038/srep41585

**Published:** 2017-01-31

**Authors:** Dimitrios Ioannou, Nicole M. Millan, Elizabeth Jordan, Helen G. Tempest

**Affiliations:** 1 Department of Human and Molecular Genetics, Herbert Wertheim College of Medicine, Florida International University, Miami, FL, USA; 2 Biomolecular Sciences Institute, Florida International University, Miami, FL, USA

## Abstract

The organization of chromosomes in sperm nuclei has been proposed to possess a unique “hairpin-loop” arrangement, which is hypothesized to aid in the ordered exodus of the paternal genome following fertilization. This study simultaneously assessed the 3D and 2D radial and longitudinal organization of telomeres, centromeres, and investigated whether chromosomes formed the same centromere clusters in sperm cells. Reproducible radial and longitudinal non-random organization was observed for all investigated loci using both 3D and 2D approaches in multiple subjects. We report novel findings, with telomeres and centromeres being localized throughout the nucleus but demonstrating roughly a 1:1 distribution in the nuclear periphery and the intermediate regions with <15% occupying the nuclear interior. Telomeres and centromeres were observed to aggregate in sperm nuclei, forming an average of 20 and 7 clusters, respectively. Reproducible longitudinal organization demonstrated preferential localization of telomeres and centromeres in the mid region of the sperm cell. Preliminary evidence is also provided to support the hypothesis that specific chromosomes preferentially form the same centromere clusters. The more segmental distribution of telomeres and centromeres as described in this study could more readily accommodate and facilitate the sequential exodus of paternal chromosomes following fertilization.

The complexity of the human genome and the interpretation of its associated functions is further compounded by the fact that chromosomes and individual regions are non-randomly organized in the 3D nuclear space. The term “genome organization” provides the framework that describes the distinct positions that chromosomes and/or specific genes occupy in the interphase nucleus^1^,^2^,^3^,^4^. Advances in microscopy and the use of fluorescence *in-situ* hybridization (FISH) have confirmed the non-random arrangement of the genome and currently chromatin organization is described as a polymer-like, fractal globule based on 3 C technology^5^. However the debate about which “theoretical” model best describes the 3D conformation of the genome is still very much under discussion^6^. Two proposed models based on the proximity patterns between chromosome territories (CTs) with regards to their nuclear position mainly describe the radial organization of CTs. The gene density model (observed in proliferating lymphoblasts and fibroblasts)^7^,^8^ positions gene-dense chromosomes toward the nuclear interior^9^; the chromosome size model (observed in quiescent, senescent, and flat ellipsoid fibroblasts) positions larger chromosomes toward the nuclear periphery^10^,^11^. The aforementioned models should not be considered as mutually exclusive, given that chromosome position depends on many dynamics that are slowly beginning to be understood. These include but are not limited to the proliferating status of the cell, the local neighborhood of the chromosome^12^,^13^, specific factors like CTCF (CCCTC-binding factor)^14^ and cohesins through their interactions to define both intricate loop structures within domains and the borders of megabase-scale chromosomal domains^15^. Recently insights on the machinery used for repositioning by genes or regions have been unveiled; including chromatin remodelers, histone modifiers, nuclear envelope and pore proteins^16^.

Apart from uncovering the molecular mechanism of topological organization of genes or genomic regions of interest, it is equally important to explore the functional significance of this organization. Several studies have shown gene repositioning relative to their respective CTs as a result of transcriptional activation during cellular differentiation^17^,^18^,^19^,^20^,^21^. Additionally, in primary fibroblasts CT reorganization for certain chromosomes (e.g. 13, 18, 10) occurs when cells enter a different replication status (e.g. quiescence)^22^. We recently showed another aspect of the plasticity of the genome when we observed CT reorganization following DNA damage with H_2_O_2_ and UVB which could be part of the DNA repair response^23^. This data together with correlations of observed alterations in the nuclear architecture with disease (e.g. laminopathies^24^, cancer^25^, diseases related to polysomies^26^, promyelotic leukemia, X-linked intellectual disabilities, Huntington disease^4^, and proximity of translocation prone regions in 3D space)^16^ suggests the requirement to maintain a stable nuclear architecture. This organization potentially confers a “healthy nuclear” state, which if perturbed could be manifested as a change in topology of a chromosome or region which could perturb normal cellular functions and responses, resulting in alterations in genomic interactions and gene expression leading to, or contributing to disease.

The spermatozoon is a unique cell that has the critical mission to transfer and propagate the paternal haploid genome to the oocyte during fertilization. The spermatozoon possesses unique features compared to somatic cells including: its small size, the presence of a flagellum for movement, its relative transcriptionally silent status, and its unique chromatin packaging. Despite the unique properties of spermatozoa the organization of chromatin in this cell type has been underexplored. Sperm chromatin is remodeled during the later stages of spermatogenesis, once the spermatogonia have committed to this pathway there is replacement of histones, initially with transition proteins (TNPs)^27^, followed by protamine replacement^28^. This ordered histone replacement allows a higher degree of compaction^29^ and condenses the nucleus to 1/13^th^ of the size of that of oocyte^30^. In humans, approximately 10–15% of the canonical histones remain bound to the chromatin^31^. The remaining histone-bound chromatin contain unique epigenetic markings postulated to be implicated in early embryogenesis development by “poising” these regions for early use in the newly formed zygote^32^,^33^.

A handful of early studies have postulated a different pattern of organization in sperm cells compared to somatic cells. These studies have assessed the radial and/or longitudinal organization of chromatin in sperm cells using FISH probes for centromeres, telomeres, locus specific regions^34^,^35^,^36^,^37^, and chromosomes^38^,^39^,^40^,^41^. Study findings have demonstrated non-random organization of targeted regions both radially and/or longitudinally. By and large, the radial intranuclear localization of sperm chromosomes seems to be driven by gene density^42^. Early studies assessing localization of centromeric sequences have revealed that chromosomes in sperm appear clustered via their centromeres to form a chromocenter in the interior of the nucleus, with the telomeres preferentially localized to the “extreme periphery” (hairpin-loop model)^37^,^39^,^43^. This hairpin-loop configuration has been proposed to have a role in the ordered “exodus” of chromosomes through their association with the sperm microtubule machinery^44^. The possible significance of sperm chromosome position (radial or longitudinal) is hypothesized to be related to a progressive exposure of certain chromosomes/chromosomal domains to the maternal ooplasm after fertilization. This ordered progressive exposure of the paternal genome could result in diverse timing of chromatin remodeling prior to pronuclear formation, transcriptional activation of the paternal genome and DNA replication^42^.

This study had several aims: i) to assess telomere and centromere organization in sperm cells from multiple normozoospermic subjects to determine if patterns of organization were reproducible; ii) to clearly delineate the number of telomere and centromere clusters observed in sperm cells and to provide detailed information on their topological localization; and iii) to investigate whether individual chromocenters are preferentially formed by specific chromosomes by assessing the localization of the nuclear organizing regions (NOR) and three individual centromeres. Study findings are discussed in the context of current proposed hairpin-loop model that has not been revisited with higher resolution methods since its inception 20 years ago^37^, along with the potential implications for fertility and early embryonic development.

## Results

The organization of the targeted genomic regions of interest (telomeres, centromeres, and NORs) was assessed by employing a FISH-based approach in ten normozoospermic males (Table 1). Radial organization was established using a 3D approach which determined the distance of the targets to the nearest nuclear edge and their localization within three distinct regions (interior, intermediate, and periphery) of the nucleus. Longitudinal organization was investigated using 3D and 2D methods to assess the distribution of telomeres and centromeres, in three different regions of the sperm nucleus (head, mid, and tail) using the sperm tail as a point of reference. To determine whether chromocenters were formed by specific chromosomes, colocalization of the pancentromere FISH probe and eight loci (NORs and centromeres 1, 5, and 19) was employed. All FISH probes utilized in this study were validated using control metaphase-spreads from lymphocytes to confirm their specificity to the targets of interest.

### Pantelomere Organization Radial – 3D

**Figure 1:**
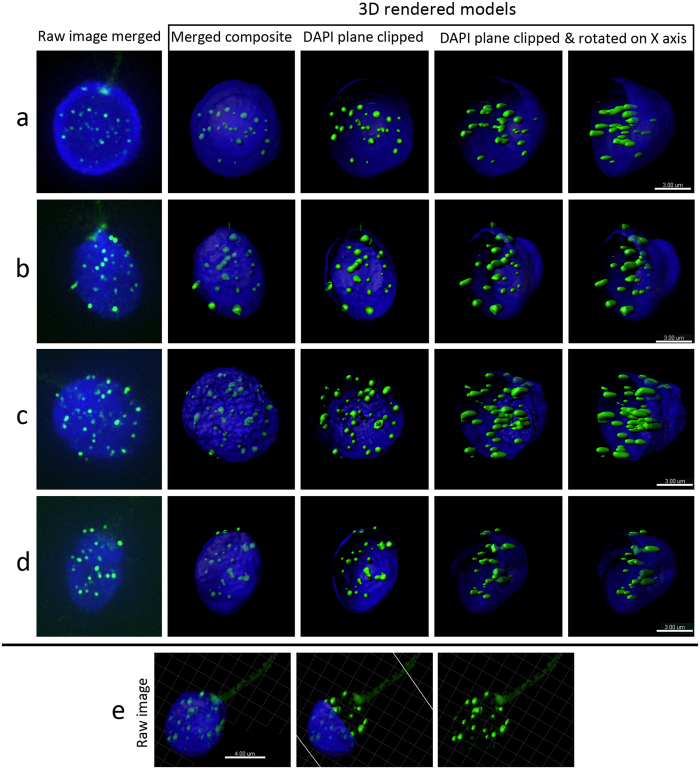
Representative images of 3D telomere organization in sperm nuclei. Panels a–d display the 3D localization of telomeres in four representative sperm cells. Nuclei are stained blue with DAPI, with telomere signals shown in green. Nuclei were captured with a Z step of 0.2 μm, with a minimum of 92 sections per nuclei using wide-field microscopy (Deltavision) and subjected to deconvolution (SoftWoRx). The first image on the left side of panels a–d, shows the raw image following deconvolution. 3D models were rendered with Imaris software (Bitplane, Switzerland, the second image in panels a–d, display the formation of 3D models with rendered telomeres (merged composite). The third image subsequently shows the same models with the DAPI partially removed (DAPI plane clipped) to better observe the 3D radial distribution of telomeres throughout all regions of the nucleus. The fourth and fifth images show the same DAPI clipped models rotated along the X-axis to further depict the 3D radial organization. Scale bar = 3 μm. Panel e displays a single sperm cell following 3D modeling that clearly depicts the sperm tail that is utilized to evaluate the longitudinal organization of telomeres with full DAPI, partially clipped DAPI and no DAPI. This clearly displays that sperm telomeres are longitudinally localized throughout the sperm nucleus from the sperm head to the tail). Scale bar = 4 μm.

**Figure 2:**
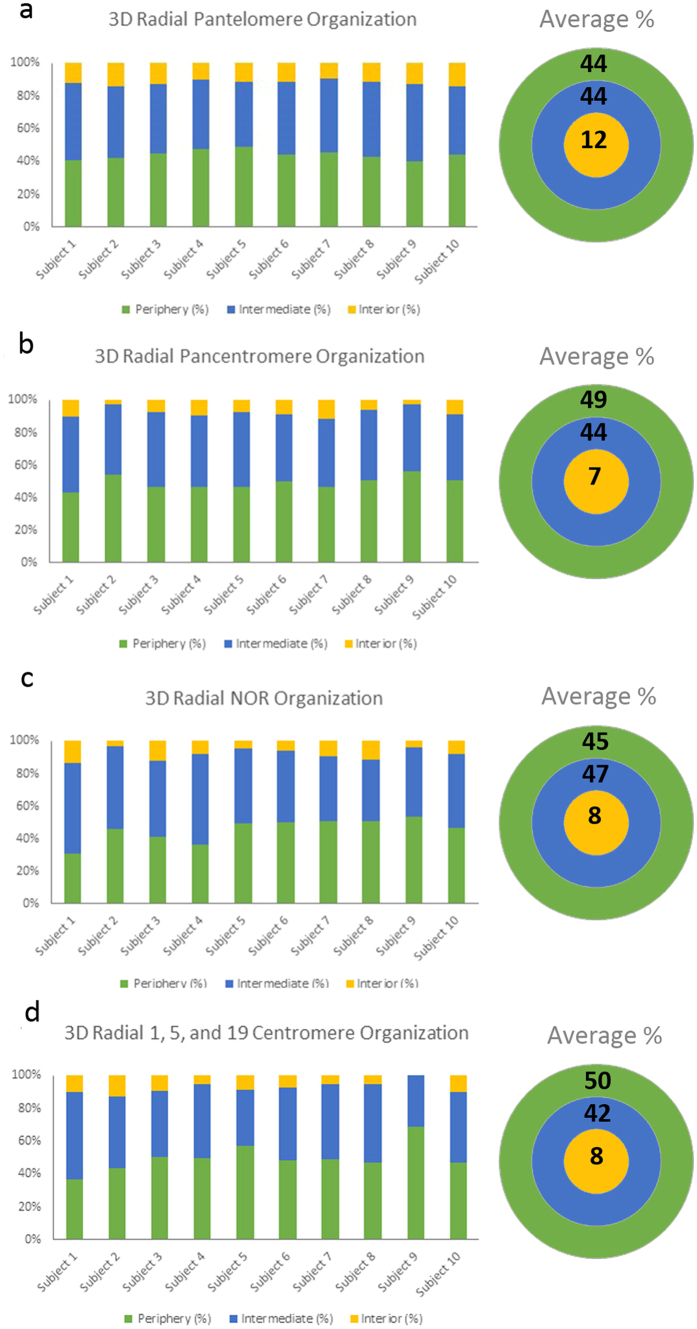
Radial organization of targeted loci in sperm nuclei. Histograms (**a–d**) display the 3D radial organization of (**a**) pantelomeres, (**b**) pancentromeres, (**c**) NORs, and (**d**) 1, 5, and 19 centromeres. The Y-axis depicts the proportion (percentage) of target loci found to be localized in the periphery (green), intermediate (blue), and interior (yellow) regions of the sperm nucleus for each of the 10 subjects, 30 cells per individual were analyzed. To the right of each histogram a schematic representation of the three regions analyzed (periphery-green, intermediate-blue, and interior-yellow) is shown. In brief, the three regions in each cell are assigned by measuring the radius of the nucleus and dividing this μm distance by three. Also displayed within the schematic model is the average distribution (percent) of the various target loci in all 10 subjects (n = 300). No significant difference in radial organization for any target loci was observed between subjects (p > 0.05).

**Figure 3:**
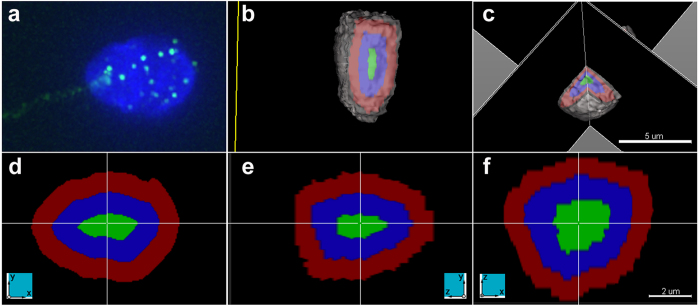
Three dimensional representation of a radially segmented sperm nucleus. Panels a-f depict different views of the same sperm nucleus. Panel a displays the raw image following deconvolution of a DAPI stained sperm nucleus with telomeres labelled in green (note the presence of the sperm tail at 8 o’clock). Panels b and c display 3D models, in which the DAPI surface depicting the nuclear periphery is pseudocolored gray. In addition, an approximation of the three radial regions: periphery (red), intermediate (blue), and interior (green) is shown within the 3D models. The radial regions are determined by dividing the sperm nucleus radius by three. Note the sperm cell has been rotated and the DAPI plane clipped at different positions to better view the radial regions (scale bar 5 μm). Panels d–f have been created using the section view tool in Imaris, which displays the same point in the data set (cross hair) along the x-, y- and z-axes simultaneously. The three radial regions (periphery red; intermediate blue; and interior green) are shown along the different axes (axes depicted in panel; scale bar 2 μm).

Non-random radial organization of telomeres was observed in the sperm from 10 subjects (n = 300; p < 0.05). Telomeres were not preferentially localized at the nuclear periphery, with telomeres distributed throughout the nucleus (Fig. 1a–d). The μm radius of the sperm nucleus was divided by three to create three distinct regions: periphery, intermediate, and interior. A schematic representation of the three radial regions is presented in Fig. 2, along with a representation of these three radial regions in 3D models (Fig. 3). The distribution of individual telomeres within these three regions was assessed using Imaris software which measures the μm distance from the geometric center of each FISH signal to the nearest nuclear edge. Each FISH signal was radially assigned to one of three regions based on the relationship between the radius of the sperm nucleus and the μm distance of the signal to the nuclear edge. The telomere localization within the sperm nucleus for all 10 subjects and the average radial organization is presented in Fig. 2a. A reproducible non-random radial telomere organization was observed in all 10 subjects (p < 0.05). The topological distribution of all telomeres from each captured image in all 10 subjects (n = 300) is presented in Supplemental Figs S1A and S1B. It should be noted that the radial analysis data is dependent on telomeres that were capable of being rendered in the Imaris software to allow the measurement of the target loci to the nearest nuclear edge. A proportion of telomeres captured (26.4%) were unable to be rendered by the software and hence were excluded from the 3D radial analysis. These signals frequently had weaker fluorescence intensity compared to the other signals, or were localized in close proximity to the sperm tail in which there was substantially more background fluorescence that did not allow individual telomeres to be isolated and rendered. It was possible to increase the signal intensity to render some of these telomeres, however, this increased telomere cluster size, often resulting in individual clusters merging. Rendering of images was performed conservatively to represent the original raw image as closely as possible, sacrificing the data for a small proportion of telomeres but retaining accuracy for the majority of telomeres.

### Pancentromere Organization Radial – 3D

**Figure 4:**
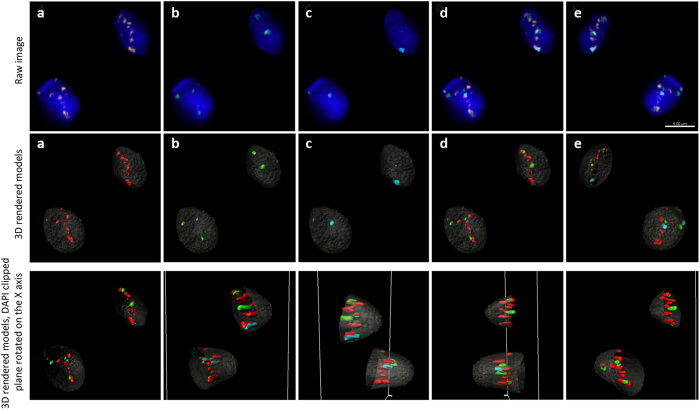
Centromere and NOR 3D organization in sperm nuclei. Panels a–e display the 3D localization of centromeres in two representative sperm cells. Nuclei were captured with a Z step of 0.2 μm, with a minimum of 92 sections per nuclei using wide-field microscopy (Deltavision) and subjected to deconvolution (SoftWoRx). The upper panel shows the raw image following deconvolution. The middle panel shows the 3D rendered models using the Imaris software, for the same cells. Nuclei are stained with DAPI (blue in the raw image; pseudocolored gray in the 3D rendered models); the pancentromeric probe staining all centromeres are red (**a**); nuclear organizing regions (NOR) are green (**b**); and centromeres 1, 5, and 19 are aqua (**c**). Image d shows the merged image of all investigated loci, and image e is the same merged image rotated 180°, showing the backside of the cell. The bottom panel, shows the same two sperm cells with the DAPI plane partially removed (clipped) and rotated along the X-axis to better observe the radial 3D distribution of centromeres and NOR throughout the sperm nucleus. Scale bar = 5 μm.

The same methodology for the pantelomere organization was utilized to assess the radial organization of centromeres in 3D for all 10 subjects (n = 300). All centromeres captured were rendered and included in the data analysis (Fig. 4). A reproducible non-random organization was found in all 10 subjects (p < 0.05), and the radial centromere organization in each of the 10 subjects and the average localization is presented in Fig. 2b. The topological distribution of all centromeres from each captured image in all 10 subjects is presented in Supplemental Figs S2A and S2B.

### NOR and Centromeres 1, 5, and 19 Radial Organization

As with the pantelomere and pancentromere probes the 3D radial organization of the NOR’s and 1, 5, and 19 centromeres was assessed utilizing the same approach. Representative FISH images of these targeted regions in individual sperm cells are presented in Fig. 4. The radial organization of NOR’s and centromeres 1, 5, and 19 demonstrated a reproducible non-random organization (p < 0.05) in all 10 subjects, and is presented for each of the 10 subjects and as an average in Fig. 2c and d, respectively.

### Pantelomere Organization Longitudinal – 3D and 2D

**Figure 5:**
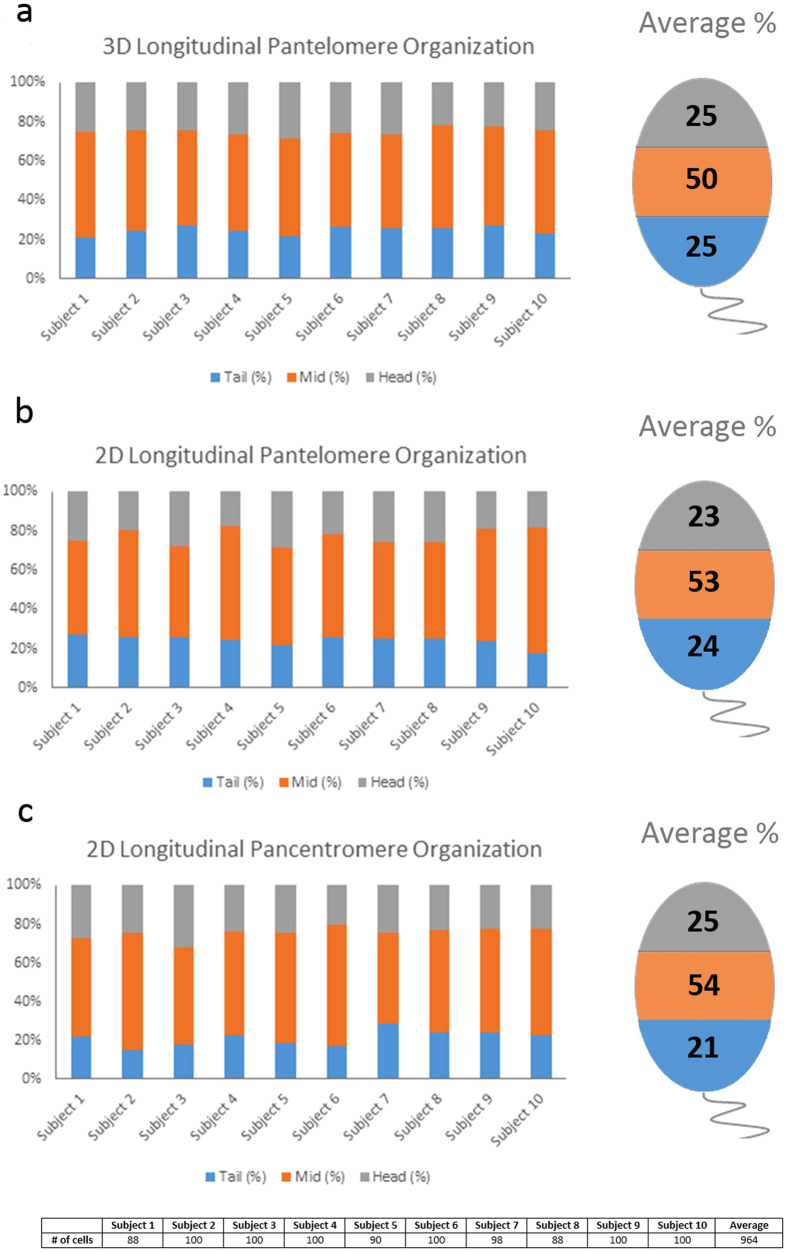
Longitudinal organization of targeted loci in sperm nuclei. Histograms (**a–c**) display the longitudinal organization of pantelomeres [(**a**) 3D; (**b**) 2D], and pancentromeres [(**c**) 2D]. The Y-axis depicts the proportion (percentage) of target loci occupying the tail (blue), mid (orange), and head (gray) region of the sperm nuclei for each of the 10 subjects, for the 3D analysis 30 cells per individual were analyzed. For the 2D analysis the number of cells analyzed per subject is listed in the table. To the right of each histogram a schematic representation of the three regions analyzed (tail-blue, mid-orange, head-gray) is shown. In brief, the three regions in each cell are assigned by measuring the length of the sperm cell from the tail to the head and dividing this μm distance by three. Also displayed within the schematic model is the average distribution (percent) of the various target loci in all 10 subjects (3D data n = 300; 2D data n = 964). No significant difference in longitudinal organization for any target loci was observed between subjects (p > 0.05).

**Figure 6:**
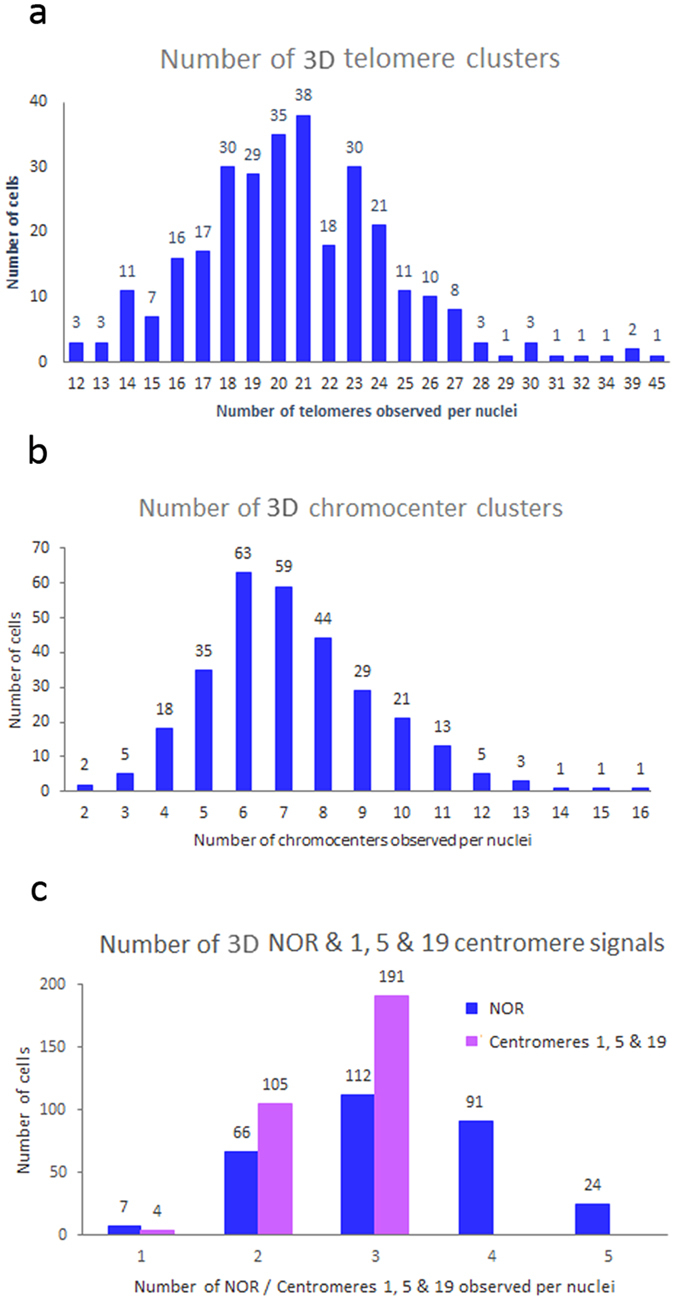
Cluster formation of targeted loci in sperm nuclei. Panels (**a–c**) display the number of individual clusters (X-axis) observed in each of the 3D sperm nuclei studied (n = 300) for telomeres (**a**), chromocenters (**b**), NOR and centromeres 1, 5, and 19 (**c**). Numbers above each of the bars represents the total number of sperm cells observed with the individual number of clusters depicted on the X-axis).

The 3D reconstructed models assessing pantelomere organization were utilized to assess the longitudinal distribution of telomeres in the sperm nucleus using the tail as a reference (Fig. 1e). The nucleus was split into three regions: head, mid, and tail by measuring the μm length from the sperm tail to the acrosomal head of the sperm cell. This length was divided by three and each telomere was assigned to one of the three regions based on which of the three segments (head, mid, or tail) each telomere was localized within^40^. A schematic representation of these three regions is presented in Fig. 5. The ability to render 3D telomeres was not a prerequisite for this method of analysis, therefore the longitudinal organization of all telomeres is included in the analysis. The same approach was used for 2D longitudinal analysis (n = 964) and the two methods were compared for coherency. The results from the 3D approach revealed a non-random (p < 0.05) longitudinal organization, the organization for each of the 10 subjects is presented in Fig. 5a. An almost identical non-random telomere organization (p < 0.05) was observed using the 2D method (Fig. 5b). The mid region contained 50.42% (3D) and 52.79% (2D) of telomeres, the tail region contained 24.81% (3D) and 24.33% (2D), with the head region containing 24.77% (3D) and 22.88% (2D). When comparing the longitudinal distribution of telomeres between the two methods (3D vs. 2D), for each reference area (head, mid, and tail) no significant differences were observed between the two methods (p > 0.05). When comparing the number of telomeres observed using 3D and 2D methods a significant difference in the number of telomeres was observed between the 3D and 2D approaches (p < 0.05). An average of 20.77 telomeres was observed per cell in the 3D analysis (Fig. 6a), of which an average of 15 telomeres could be rendered. The 2D approach revealed an average of 12 telomeres per cell. The 2D and 3D results provide evidence that telomeres aggregate to form clusters, and suggest that the 2D method utilized was not able to accurately detect all telomeres.

### Pancentromere Organization Longitudinal – 2D

To assess the longitudinal organization of centromeres data was only obtained from 2D analysis. This was due to the inability to visualize the majority of sperm tails in the 3D centromere FISH experiments due to less fluorescence background from the FISH probes, thus, no reference was available to define with certainty the head-tail axis. The longitudinal analysis of the centromeres was assessed using the same 2D approach as described for the telomere longitudinal analysis. The 2D longitudinal distribution of centromeres demonstrated a statistically significant non-random reproducible organization for each of the ten subjects (n = 964) (p < 0.05), data shown in Fig. 5c. Both the 3D and 2D pancentromere results provide evidence of centromeres aggregating to form clusters or “chromocenters”. No significant difference was identified between 3D or 2D methods regarding the number of centromeres observed per cell (p > 0.05). With the 3D approach an average of 7.22 chromocenters (Fig. 6b) was observed, versus an average of 7.46 chromocenters with the 2D approach.

### NOR and Centromeres 1, 5, and 19 Chromocenter Colocalization

In order to determine whether chromocenters were preferentially formed by the same chromosome centromeres, several different loci were cohybridized with the pancentromere FISH probe (Fig. 4). Specifically, a centromere probe that shares sequence homology with three centromeres (1, 5, and 19), and a probe that hybridizes to the NOR. The NOR include sequences in close proximity to the centromeres of the acrocentric chromosomes (13, 14, 15, 21, and 22). The number of discrete signals visualized for the NOR and centromeres 1, 5, and 19 to be physically colocalized or in close proximity to individual chromocenters in 3D was recorded in all 10 subjects (n = 300). If the targeted regions (NOR and centromeres 1, 5, 19) each contributed to the formation of different chromocenters, eight discrete signals would be expected. However, if these regions clustered to form the same chromocenters fewer discrete signals colocalized to chromocenters would be observed. In terms of discrete signals, an average of 3.2 NOR signals and 2.6 signals from centromeres 1, 5, and 19 were visible per cell in the 10 subjects (Fig. 6c). The NORs predominantly associated with three to four different chromocenters (>67%). A single, or five distinct NOR signals was rarely observed in sperm cells (<10%). When examining individual centromeres (1, 5, and 19), it was rare to observe these three centromeres forming a single chromocenter (<2%), rather they predominantly associated with three different chromocenters (>63%). Analysis of the colocalization of centromeres (1, 5, or 19) and the NOR chromosomes revealed that they formed the same chromocenter in less than 25% of cells.

### Study Limitations

Sample preparation, FISH procedure, image capturing, deconvolution, 3D model rendering, and data analysis may all potentially contribute to small variations between cells and experiments. However, these variables were minimized by ensuring all of these steps for the 3D aspect of this study were performed by the same experienced operator following the same procedures and through the use of established and accepted methodologies^45^,^46^,^47^,^48^,^49^. Similar approaches to the longitudinal method of analysis performed in this study has been previously published by several groups^39^,^40^,^50^,^51^. This approach was modified to provide data on the radial organization which utilized a Euclidean Distance Transform (DT) to measure distance of the geometric center of target loci to the nearest nuclear edge to assign target loci to one of three radial regions. The DT is shape invariant and does not impose an assumption on the shape of the cell. Nevertheless, utilization of the DT to calculate the relative radial position is appropriate for regular shaped nuclei such as ellipsoid sperm cells^49^. To facilitate comparisons across experiments, DT measurements were normalized against the widest radial diameter of individual sperm nuclei accounting for size differences between sperm nuclei. The normalized DT provides a rough correction for nuclear size, and thus is not directly comparable for objects of different shape^45^,^47^. However, due to the fact that human sperm cells are similar in size and shape this has minimal consequences in the current study. The radial and longitudinal length of individual sperm were used to segment the nucleus into three regions interior, intermediate, and periphery for the radial analysis and head, mid, and tail for the longitudinal analysis. These regions are created through the division of the sperm cell nucleus μm radius or μm length respectively. Thus these regions are not of equal volume (3D) or area (2D), and do not account for variations in the contours of the nucleus. Furthermore, it is important to note that the volume of each 3D radial segment (Fig. 3) was not possible to determine, and the radial segments illustrated in the 3D Fig. 3 models only provide a visual approximate representation of the 3D segmentation of the nucleus. However, size differences between cells are incorporated in this approach as the radial and longitudinal measurements are performed for individual cells. It is also important to note that the same approach was utilized for all cells to assign the three regions radially and longitudinally and revealed highly reproducible organization between all subjects for all targeted loci radially and longitudinally within the defined regions. Furthermore, both 2D and 3D longitudinal analysis of the telomeres provided highly reproducible findings. Additionally, utilizing the same 3D radial approach in lymphocytes we provided reproducible 3D organization of chromosome territories in lymphocytes and the chromosome organization pattern observed corroborated those reported by an independent laboratory utilizing widely accepted 2D methodology for examining genome organization^7^,^52^. Furthermore, using this 3D methodology we were able to measure chromosome repositioning following global transcriptional activation with phytohemagglutinin^52^. Importantly the Imaris software utilized, measures the μm distance of the geometric center of each FISH signal to the nearest nuclear edge providing an accurate localization of each target loci within the nucleus. Taken collectively, the data suggests that the current methodological approaches can be utilized to assess nuclear localization, reproducibility between subjects, and can be used to measure repositioning. Additionally, a proportion of telomeres typically localized in close proximity to the sperm tail, or with weaker signal intensity were unable to be rendered by the Imaris software to assess their radial distribution, and hence were excluded from the radial analysis. However, even if all the non-rendered telomeres were localized in the peripheral region, this would result in a radial telomere distribution from the periphery, intermediate, and interior regions of 57%, 33%, and 10%, respectively. These results still reflect that over 40% of telomeres are not found within the nuclear periphery. From the current study it was also not possible to determine whether the same NORs or individual centromeres investigated (1, 5, or 19) preferentially formed a single chromocenter.

## Discussion

**Figure 7:**
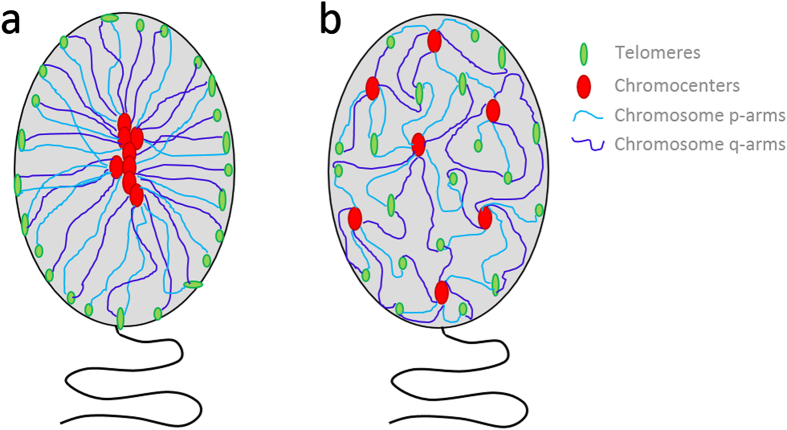
Schematic representation of the proposed models of chromatin organization within sperm nuclei. Panel a presents a schematic representation of the hairpin-loop model in which there is a linear array of chromocenters (red), with the chromosome p- and q-arms (light blue and dark blue, respectively) stretching out toward the telomeres (green) which are localized at the “extreme periphery” of the nucleus. In panel b we present a refined version of the model which depicts a more segmented organization, with telomeres and chromocenters being localized throughout the nucleus. Upon fertilization, chromatin localized at the apical most region and situated near the nuclear envelope will be the first regions of the genome to enter the oocyte and be remodeled.

Non-random organization within the sperm nucleus is an accepted concept that has been proposed to have potential functional implications for fertilization and early embryogenesis. The current spatial organization model postulates that all chromosomes aggregate through their centromeres in the center of the nucleus forming a chromocenter which is made of pericentric heterochromatin, with the telomeres localized at the “extreme periphery”, forming a hairpin-loop configuration (Fig. 7a). However, the core components of the hairpin-loop model were described utilizing lower resolution microscopy and imaging, have not been extensively reevaluated since it was conceptualized over 20 years ago^37^. Additionally, most published studies have several major drawbacks that hinder study replication (e.g. provision of limited description of the methodology utilized; utilize an undeterminable number of cells; do not provide detailed semen parameters or detailed information regarding the topological organization of targeted regions within sperm)^35^,^37^,^38^,^43^,^53^. Telomeres are important features of chromosomes conferring stability, genome integrity, and possess crucial biological features (e.g., length) for human reproduction^54^,^55^,^56^. Their spatial role in the sperm nucleus has been postulated to aid in the sequential exodus of chromosomes during fertilization and these regions are hypothesized to be among the first responders to oocyte signals to begin the formation of the male pronucleus^42^,^44^,^57^, a feature that bears an epigenetic characteristic for the onset of embryonic development. It is important to note that this hypothesis originates from observations in mouse embryos and has not yet been validated in humans^58^. Similarly, it has been proposed that centromeres of non-homologous chromosomes are arranged to form a chromocenter, which is hypothesized to play a fundamental role in proper intranuclear chromosome positioning to determine chromosome localization^53^.

The radial and longitudinal organization of all targets (telomeres, centromeres, and NORs) was non-randomly organized and highly reproducible among the 10 normozoospermic subjects enrolled in this study. The results from this study confirm the presence of telomere clusters, forming dimers or tetramers^37^,^59^,^60^. However, in contrast to early studies, the current study provides evidence that not all telomeres were localized in the “extreme periphery”. Rather, data suggests a more segmented telomere organization that is highly reproducible between subjects. The longitudinal organization of telomeres (3D and 2D) provides further evidence of a more segmental organization of telomeres (Fig. 7b), with a significant proportion of telomeres clustering to form a “belt” in the mid part of the sperm nucleus, a result that corroborates initial observations^37^.

Initial studies proposed a single interiorly localized chromocenter^37^, the chromocenter was latterly described as a linear array of centromeres^39^. The existence of multiple chromocenters has been confirmed by a recent 3D study. This study reported fewer than 10% of spermatozoa containing a single chromocenter, with 73% of cells exhibiting 1–3 chromocenters^53^. In the current study, no nuclei were found to contain a single chromocenter, with an average of 7 chromocenters observed per cell (3D and 2D). Furthermore, in contrast to previous studies evidence is provided to suggest centromeres are preferentially localized in intermediate and peripheral localizations (~92%). The data suggests that centromeres cluster to form multiple chromocenters that display a more segmented organization occupying discrete locations, which are not bound to the interior region of the nucleus (Fig. 7b). This more segmented organization of chromocenters may be crucial for the ordered exodus of paternal chromosomes following fertilization. If this is the case, it stands to reason that each chromocenter may be preferentially composed of the same chromosomes. To address this, eight different regions (NORs and centromeres 1, 5, and 19) were studied to determine if they preferentially formed specific chromocenter clusters more frequently than would be expected by chance. It is accepted that the NOR forms a cohesive cluster in the nucleolus of somatic cells^53^ and evidence of clustering was suggested in a previous report in sperm cells^61^. However, in this cohort NORs predominantly formed three-to-four discrete loci. The observation of only partial clustering for the NORs is in agreement with the concept described as the unlocalized structural element^61^, which could be an important reflection of the silent role of NOR in sperm until the first stages of embryo development^50^,^53^. Additionally, centromeres 1, 5, and 19 rarely formed the same cluster, and infrequently was a single chromocenter cluster found to contain a NOR with any of the targeted centromeres. Although these findings cannot confirm the hypothesis that chromocenters are preferentially composed of the same chromosomes, the aforementioned findings occurred more often than would be expected by chance alone. Future studies will further examine this by co-hybridizing different combinations of individual centromeres with the pancentromere FISH probe.

Radial and longitudinal assessment using 3D methods provide a more precise physical localization and allow better visual representation of the organization of target loci within the nucleus. However, 2D methods have several advantages in that they are rapid, allow significantly more cells to be analyzed, are less labor intensive, and do not require superior computing and imaging software for analysis. Previous studies have suggested that 2D radial organization yields comparable results compared to 3D methods^22^,^52^. However, 3D and 2D methods revealed significant differences in the number of telomere clusters observed but no significant difference was observed for the centromeres. The findings from this study suggest that 2D and 3D methods may yield different results when investigating multiple loci (>8), thus when examining multiple loci 3D methods should be utilized.

In summary our findings were reproducible amongst the ten normozoospermic subjects, revealing that not all telomeres are localized at the “extreme periphery”, with multiple chromocenters localized throughout the nucleus. Furthermore, we provide indirect evidence that individual chromocenters may be preferentially formed by specific chromosomes which likely determines chromosome position within the sperm nucleus, both radially and longitudinally. Taken collectively, these findings suggest the proposed hairpin-loop model needs to be further redefined to take into account the more segmental radial and longitudinal organization found throughout the sperm nucleus in this study (Fig. 7). The reproducible segmental organization observed in the sperm nucleus likely serves several critical functions including: i) to efficiently compact the paternal DNA to protect the genome from DNA damage, and to assume the required nuclear size and shape for successful passage through the female reproductive tract; ii) to effectively silence gene expression, but allow rapid reactivation post-fertilization; and iii) to deliver the paternal genome in a sequential order to allow the maternal oocyte to efficiently repair DNA damage and remodel the paternal chromatin, critical for early embryonic divisions^61^. Additionally, sperm-FISH studies have shown that whole chromosomes are preferentially ordered along the head-tail axis^35^,^38^,^39^,^40^,^50^,^53^, which provides further evidence to support the more segmental distribution of telomeres and centromeres described in this study. This more segmented organization could more readily accommodate and facilitate the sequential exodus of paternal chromosomes following fertilization. Perturbations in this nuclear organization could disrupt the critical sequence of events required for pronuclear formation and early post-zygotic development. Further studies are needed to determine whether specific chromosomes (and genes) critical for early embryogenesis occupy specific nuclear regions in the sperm nucleus. Future studies are also required to establish whether perturbations in this organization can be identified between fertile and infertile men. These studies should primarily focus on identifying couples undergoing assisted reproductive techniques that experience recurrent fertilization failure and early embryonic arrest with no obvious contributory female factor.

## Materials and Methods

### Patient cohort and semen analysis

This study was approved by the Florida International University Institutional Review Board (FIU-IRB-13–0044). All methods were carried out in accordance with the approved guidelines. Ten normozoospermic individuals undergoing routine semen assessment at IVF Florida Reproductive Associates in Margate, Fl, USA all provided written informed consent to participate in this study. The average age of the individuals was 35.9 years (ranging from 29–48). Samples were collected via masturbation; and were classified as normozoospermic based on the World Health Organization criteria^62^ semen parameter guidelines (Table 1). The remainder of the semen sample surplus to the semen parameter assessment was snap frozen and stored in liquid nitrogen until further use.

### Sample preparation

The detailed method described in ref. 40 was used to prepare the semen samples for FISH. Briefly, samples were thawed at room temperature and washed with 10 mM NaCl −10 mM Tris (pH 7.0) followed by centrifugation for 7 minutes at 1,900 rotations per minute (rpm). After removal of the supernatant, the pellet was resuspended in the same buffer, and cycle was repeated 3–5 times depending on the pellet size to remove the seminal fluid. The sample was then fixed using 3:1 methanol:acetic acid to a final volume of 5 ml following centrifugation (1,900 rpm – 7 minutes). Following removal of the supernatant the cycle was repeated depending on the pellet size to a maximum of 5 times. An amount of 1–3 μl was placed on a glass slide (Fisher Brand^®^) and evaluated under a differential interference contrast microscopy (Olympus BX53) for optimal cell density.

### Sperm fluorescence *in-situ* hybridization (FISH)

A similar FISH approach described in ref. 52 was used with the following differences. No pepsin treatment was utilized to retain sperm flagellum. Additionally, prior to initiating the aforementioned FISH approach, a 20 minute incubation in 0.1% DTT (Sigma Aldrich, St Louis, MO, USA), 0.1% Tris solution (pH 8.0) in the dark, was utilized to swell the sperm cells to allow the FISH probes to access and enable incorporation into the DNA. This step was followed by a rinse with 2 × saline sodium citrate (SSC; Fisher Scientific, Pittsburgh, PA, USA), before dehydrating with ethanol (70–80–100%) and moving to paraformaldehyde fixation. To investigate the positioning of all telomeres and/or centromeres a Pantelomeric (Cat No 1696; labelled in green) and Pancentromeric (Cat No 1695; labelled in red) probe from Star*FISH© (distributed by Cambio Cambridge, UK) was utilized as per the manufacturers guidelines. Hybridization occurred for approximately 16 hours at 37 °C in a Thermobrite^®^ Statspin (Abbott Molecular, Illinois, IL, USA). Post hybridization washes were as per the manufacturers guidelines with the addition of a ddH_2_O (double distilled H_2_O) rinse for 1 minute and an ethanol series (70–80–100%). Following these washes, slides were air dried and subsequently mounted with 4′,6-diamidino-2-phenylindole (DAPI – Vector Labs, Burlingame, CA, USA) under a 24 × 55 mm coverslip. To investigate chromocenter formation FISH probes from Kreatech (distributed by Leica Biosystems, Illinois, USA) specific to the NOR and three centromeres (1, 5, and 19) that shared sequence homology were hybridized with the pancentromeric probe. The following probe sets were used: pKI-20033G (p-arm of all acrocentric chromosomes) in green, pKI-20026B (targeting pericentric heterochromatin sequences) for 1, 5, and 19 centromeres in blue, pKI-2000R (pancentromeric probe) labelled in red. The protocol used was as described above^52^ with the following alteration regarding probe preparation and hybridization conditions. A 1:1:1 ratio mix of all 3 probes was denatured at 73 °C for 10 minutes and subsequently co-denatured with sperm cells for 90 seconds in a Thermobrite^®^ Statspin (Abbott Molecular, Illinois, IL, USA) followed by hybridization for a minimum of 16 hours at 37 °C. Swelling of cells should be avoided for all organization studies, but due to the unique sperm DNA packaging it is a prerequisite for sperm-FISH. This step is critical to monitor to cause minimal disruption to the nuclear organization, therefore experiments were performed to select decondensation conditions that provided efficient FISH hybridization (>95%) and resulted in minimal reproducible swelling between samples. Imaging software Imaris (V.7.6.3 Bitplane – Zurich, Switzerland) was utilized to calculate the volume of the DAPI sperm cells following the same FISH procedure (minus DTT swelling) and sperm cells following FISH with various DTT concentrations. The decondensation parameters above, provided mild reproducible decondensation resulting in an average 2.4 fold DAPI volume increase for the DTT treated sperm nuclei versus untreated sperm nuclei (average nucleus volume 153.9 μm^3^ versus 62.83 μm^3^, respectively), this volume increase is lower than previously reported literature^53^. Additionally, all FISH probe sets were initially tested on human metaphase chromosomes and >25 metaphase spreads were karyotyped to ensure FISH probe target specificity.

### Image acquisition (2D) and longitudinal organization assessment

An Olympus BX61 epifluorescence microscope equipped with a cool charged coupled device (CCD) camera (Hamamatsu ORCA – R2 C10600) and a motorized ES111 Optiscan stage (Prior Scientific UK) was used to capture all images regarding the 2D aspect of this study. Three single band pass filters for FITC, TRITC, and DAPI (Chroma Technology, Bellows Falls, VT, USA) were used. All images were acquired using Smart Capture 3.0 (Digital Scientific, UK) exported as.tiff files for further analysis. A minimum of 100 cells when possible, were analyzed per subject and probe set. The longitudinal analysis, was performed as per previous studies^40^. In brief, the distance from the point of tail attachment towards the head was measured in Image J for each sperm cell. The cell was then divided into three areas (based on the tail-head distance) and the distribution of each signal was assigned to the head, mid, or tail region of the sperm cell. Schematic representations of the three distinct areas is depicted demonstrating the average percent localization of target loci in Fig. 5(b,c). The Chi-squared goodness of fit (χ^2^) was utilized to evaluate if the organization of each target of interest differed from random (p < 0.05).

### Image acquisition (3D), radial and longitudinal organization assessment

The recently published methodology^52^ was utilized to capture images in 3D using DeltaVision (Applied Precision, WA, USA) imaging station; consisting of an Olympus IX71 inverted microscope with 60X, 1.4 NA oil-immersion lens and a photometric CCD. All images were taken with a Z step size of 0.2 μm (92 optical sections), saved as 3D stacks and subjected to constrained iterative deconvolution using the same standard settings (DeltaVision – SoftWoRx -V 5.5; Applied Precision, WA, USA). A minimum of 30 images per subject and probe set were acquired using TRITC (594 nm), FITC (523 nm), CFP (480 nm) filter combinations depending on the FISH probes utilized. 3D reconstruction of the captured images (as 3D stacks) from SoftWoRx were rendered in 3D and analyzed using the Imaris software (V.7.6.3 Bitplane – Zurich, Switzerland) by converting images to 32-bit float images. The DAPI nuclear periphery in the 3D model was rendered by creating a surface in the Imaris program, an iso-surface was created to visualize the object in 3D space, allowing verification of the accuracy directly against the original raw image. The surface creation was defined by setting an intensity threshold to select the voxels that were considered part of the reconstructed iso-surface. This threshold required minimal manual user intervention at times, to best reflect the raw data set, and to remove background fluorescence. Additionally, voxel selection was enhanced by applying a Gaussian filter prior to selection, to remove noise and signal that was not attributed to the labelled structure. This smoothing step adjusts for the limits of resolution due to the acquisition system, and quality of the tissue/labelling and ensures that the voxels from “out of focus light”, which typically appears as a background blur in the Z-plane, was not overly selected as part of the surface structure ensuring a true representation of the sperm nucleus. The reconstructed DAPI surface was overlaid on the raw image to ensure the surface fitted the raw data in all 3 axes. To measure the nuclear localization of target loci within the nucleus the DAPI surface object denoting the nuclear periphery was used as a region of interest to isolate other channels (FISH signals) within the nucleus. This masking process generated a new channel based on the voxels that lie inside of the 3D volume of nuclear periphery for each individual fluorochrome. This new channel was rendered without interference from other voxels in the dataset, creating a new surface segmentation of structures within the nucleus. The Imaris Distance Transform (DT) Matlab XTension utilizes a 3D quasi-Euclidean distance transform from the geometric center of each rendered FISH signal in the data set to the binary mask of the DAPI border of the surface (nuclear periphery). The DT calculates the shortest distance in 3D space between each data point (FISH signals) and the DAPI nuclear periphery (surface border) with minimal user intervention, following the contours of the nucleus^63^,^64^,^65^,^66^. The DT is shape invariant and does not impose any assumptions on the cell shape or size^45^. However, to facilitate comparisons across experiments, it is desirable to have a measure which is both scale and shape invariant; thus the DT measurements were normalized against the widest “radial” diameter (90° to the tail) of individual sperm nuclei to account for differences in the sizes of individual nuclei. Based on the DT measurements, signals were individually assigned to one of the three areas (interior, intermediate or peripheral) using as reference the radius of the cell. A schematic representation of the three areas is presented in Fig. 2(a–d), along with an approximate representation of these regions in 3D models (Fig. 3). A similar approach as described for the 2D analysis was used to determine the 3D longitudinal arrangement of telomeres, whereby the normalization occurred against the diameter from the point of tail attachment in the sperm cell to the head and telomeres were individually assigned into one of the three regions (tail, mid, and head), see Fig. 5a for schematic representation of the three areas. The Chi-squared goodness of fit (χ^2^) was utilized to evaluate if the organization of each target of interest differed from random, a p-value of <0.05 suggested a non-random distribution. For the observation of any interactions (e.g. NOR with specific centromeres or chromocenters) the 3D rendered models were used, and the number of discrete signals and interactions occurring was recorded for each cell. Although both 2D and 3D approaches can be used to address the same problems, they utilize different approaches allowing the investigation of alternate aspects of topology. To compare 3D and 2D data, a two-tailed paired t-test was used, a p value < 0.05 would indicate a significance difference between the two methods.

## Additional Information

**How to cite this article:** Ioannou, D. *et al*. A new model of sperm nuclear architecture following assessment of the organization of centromeres and telomeres in three-dimensions. *Sci. Rep.* **7**, 41585; doi: 10.1038/srep41585 (2017).

**Publisher's note:** Springer Nature remains neutral with regard to jurisdictional claims in published maps and institutional affiliations.

## Supplementary Material

Supplementary Figures 1 and 2

## Figures and Tables

**Table 1:** Semen parameters for the 10 subjects enrolled in this study.

Subject	Age	Concentration (10^6^/ml)	Motility (%)	Progressive Motility (%)	Normal Forms (%)
1	32	125	96	80	4
2	33	60	87	80	4
3	43	137	80	76	6
4	48	133	98	92	5
5	30	70	91	87	5
6	42	66	68	60	10
7	29	92	78	60	6
8	38	48	73	57	4
9	29	50	90	66	4
10	35	48	59	54	4
**Mean**	**35.9**	**82.9**	**82**	**71.2**	**5.2**
*WHO guidelines*	*—*	≥*15*	≥*40*	≥*32*	≥*4*

As per the 2010 World Health Organization (WHO) semen parameter guidelines^62^ all subjects were normozoospermic.
