# Supplementary material for: A new model of sperm nuclear architecture following assessment of the organization of centromeres and telomeres in three-dimensions
Source: Sci Rep. 2017 Jan 31;7:41585. doi: 10.1038/srep41585 (PMC5282497; doi:10.1038/srep41585)
Supplement: Supplementary Figures 1 and 2 [file srep41585-s1.pdf]

A new model of sperm nuclear architecture following assessment of the organization of centromeres and telomeres in three-dimensions

Dimitrios Ioannou [1], Nicole M. Millan[1], Elizabeth Jordan[1], Helen G. Tempest [1, 2 *]

[1] Department of Human and Molecular Genetics, Herbert Wertheim College of Medicine, Florida International University, Miami, FL, USA

[2] Biomolecular Sciences Institute, Florida International University, Miami, FL, USA

* Corresponding author Helen G. Tempest, htempest@fiu.edu

Supplemental Fig S1

Micrometer distance of telomeres from the DAPI nuclear edge in the spermatozoa from 10 subjects

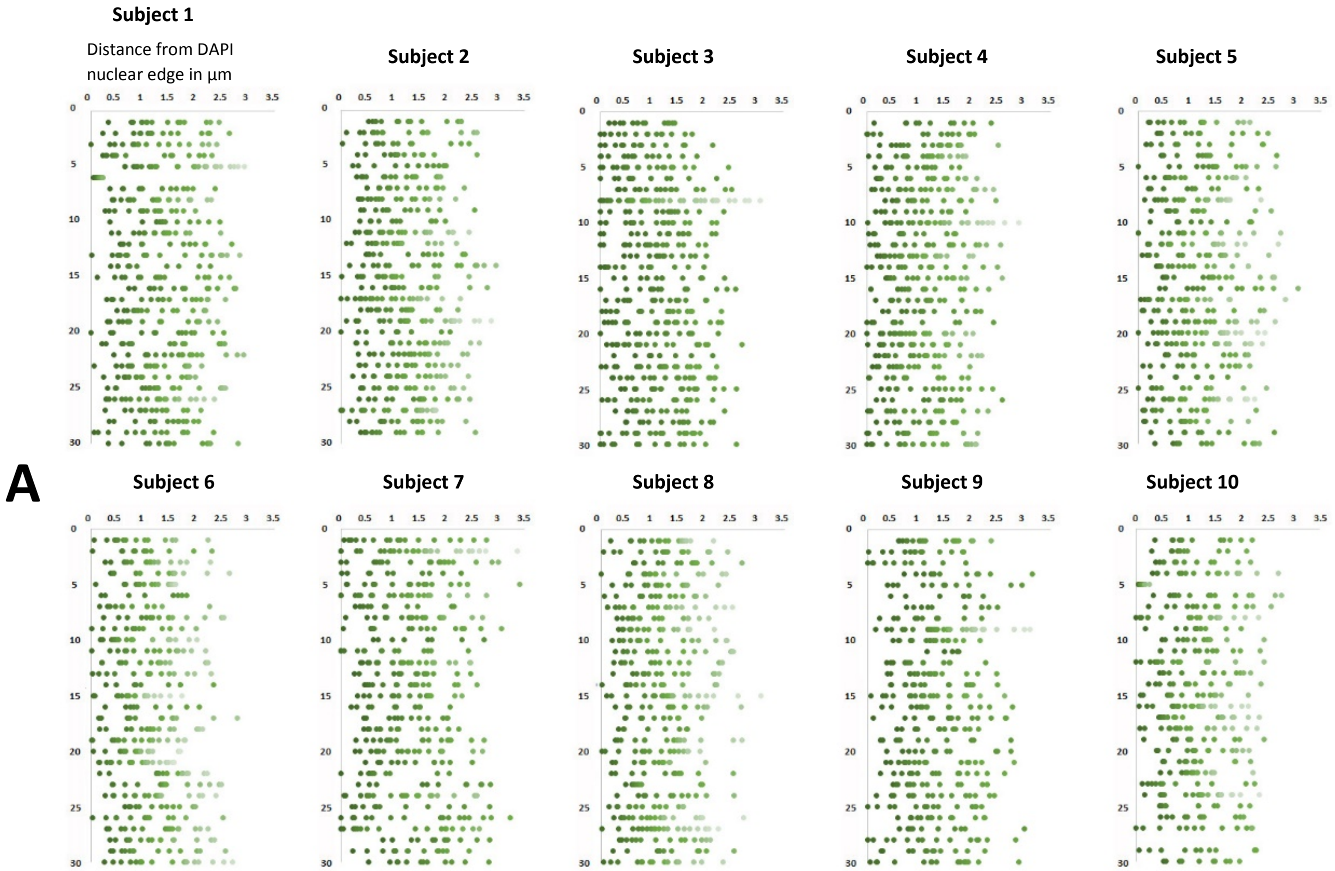

Normalized 3D radial organization of telomeres from the nuclear periphery to the interior in 10 subjects

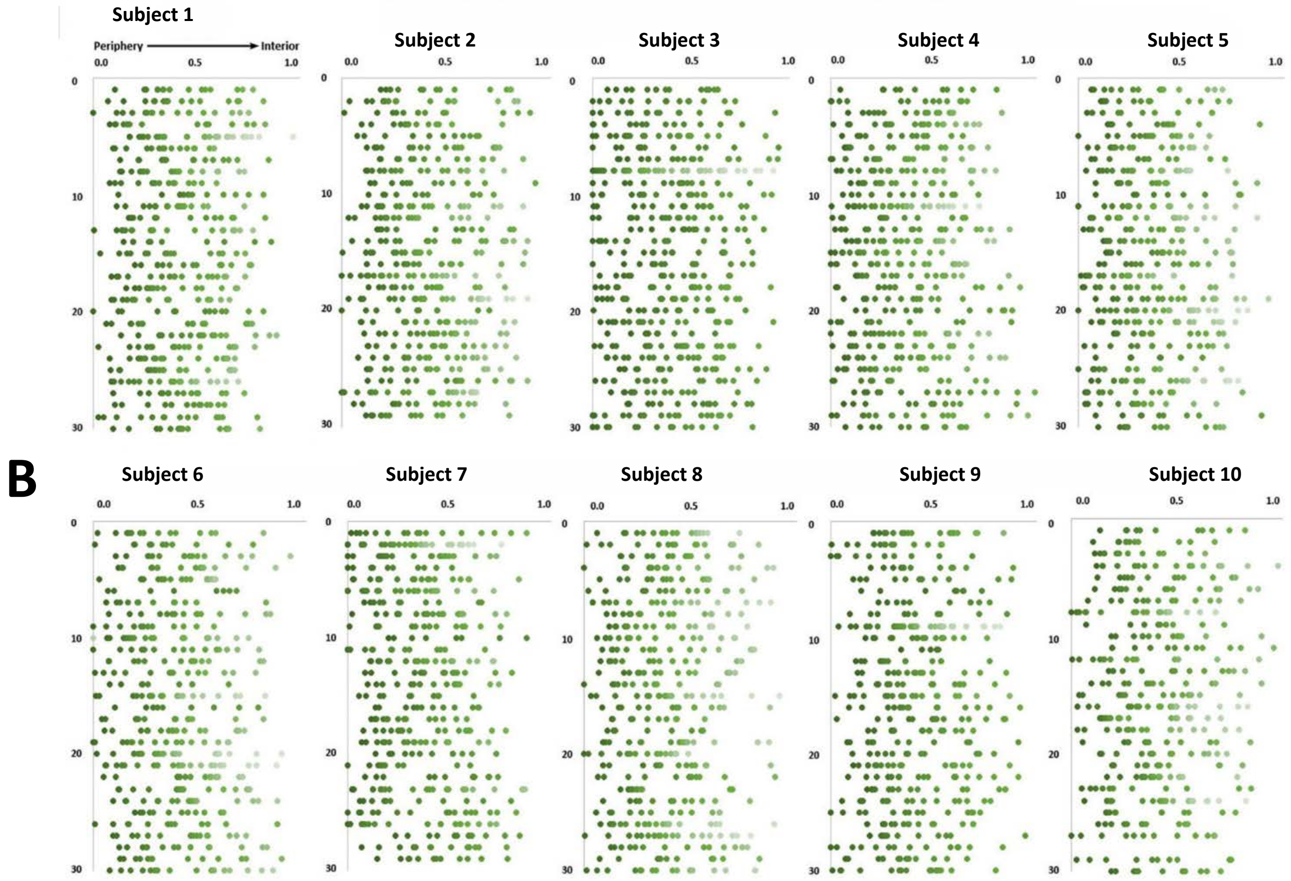

Supplemental Fig S2

Micrometer distance of centromeres from the DAPI nuclear edge in the spermatozoa from 10 subjects

A

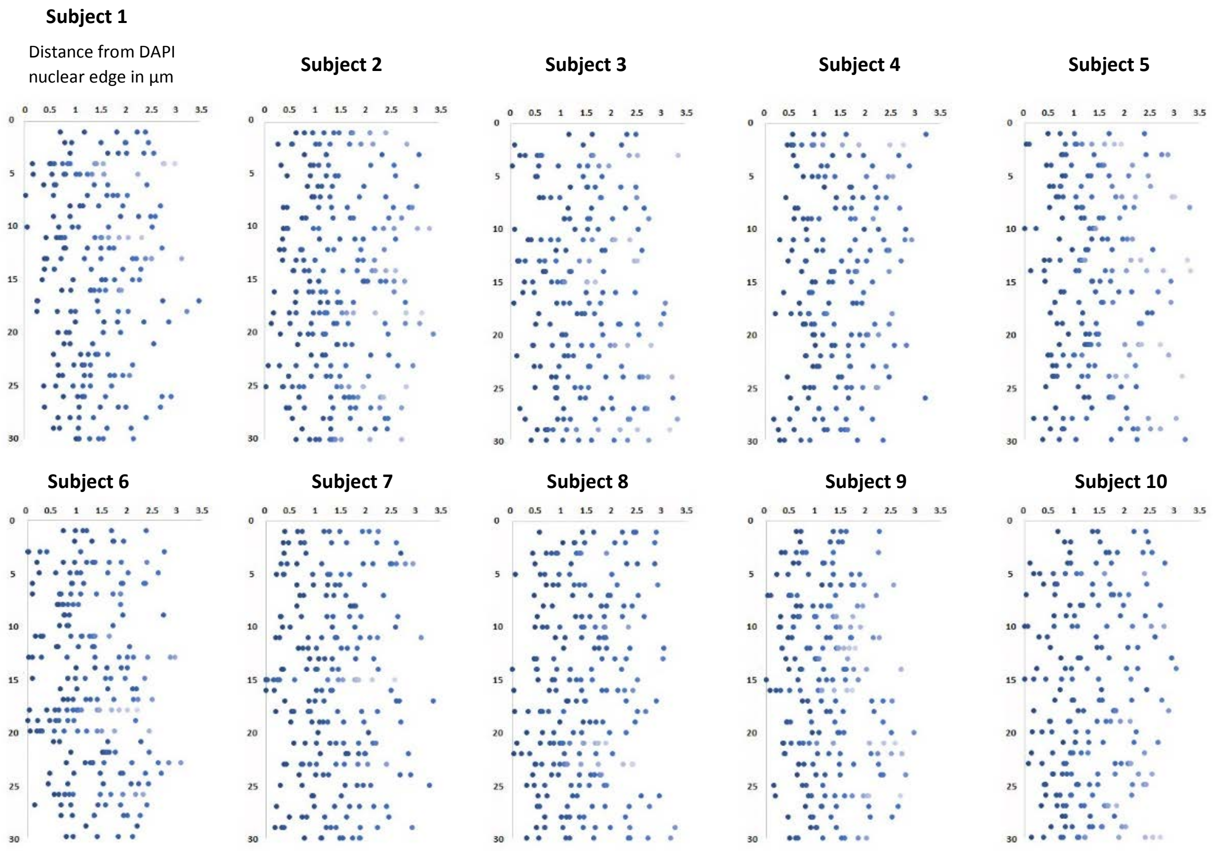

Normalized 3D radial organization of centromeres from the nuclear periphery to the interior in 10 subjects

B

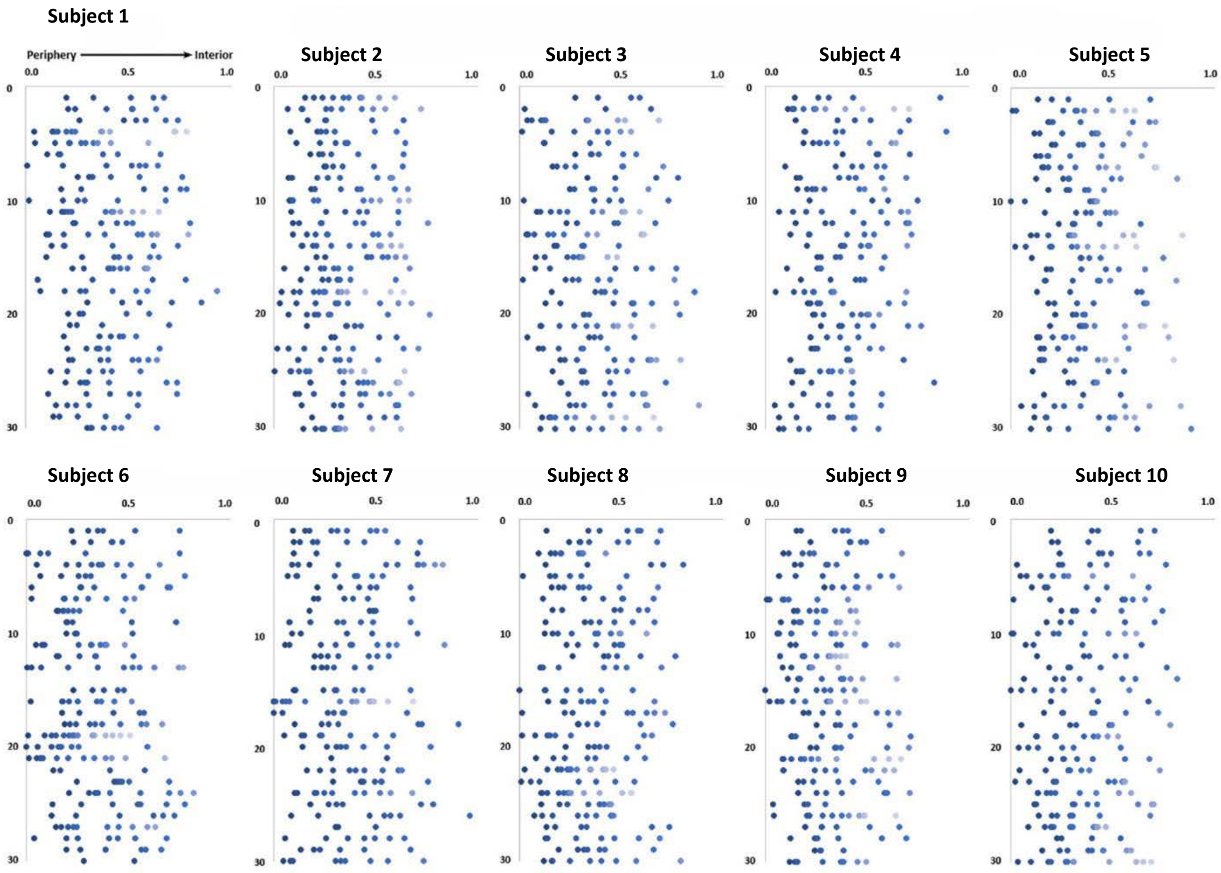

Supplemental Figures Legend

The data presented in Supplemental Figs S1 & S2 displays the 3D radial localization of each telomere (Fig S1), and centromere (Fig S2) from the 10 subjects studied. Each histogram represents a single subject and displays the radial organization (X-axis) for each of the 30 cells studied per subject (Y-axis) (total n=300). Each dot in the histogram represents the radial localization of a single telomere (green) or centromere (blue), or an individual cluster of telomeres or centromeres.

Supplemental Figs S1A and S2A displays the distance of each 3D modeled telomere (S1A) and centromere (S2A) in micrometers from the nearest nuclear edge (X-axis).

Supplemental Figs S1B and S2B displays the same data, however the distance has been normalized by dividing each micrometer measurement to the nearest nuclear periphery by the nuclei diameter of each individual cell. The nuclear periphery is depicted on the X-axis as 0.0 (left-side) and the center of the nucleus as 1.0 (right-side).

Thus telomeres or centromeres in Supplemental Figs 1A, 1B, 2A, or 2B localized toward the nuclear periphery or the nuclear interior would be found toward the left- or right-side of the histogram, respectively. Each row of dots (left-right) depicts the precise nuclear localization of all telomeres/centromeres observed in a single cell. The Y-axis (0-30) displays the data obtained for each of the 30 cells studied per subject.
